# Attentional selection is a sufficient cause for visual working memory interference

**DOI:** 10.1167/jov.23.7.15

**Published:** 2023-07-24

**Authors:** Zachary Hamblin-Frohman, Stefanie I. Becker

**Affiliations:** 1 School of Psychology, The University of Queensland, Brisbane, Australia; 2 School of Psychology, The University of Queensland, Brisbane, Australia

**Keywords:** visual search, attention, visual working memory, selection

## Abstract

Visual attention and visual working memory (VWM) are intertwined processes that allow navigation of the visual world. These systems can compete for highly limited cognitive resources, creating interference effects when both operate in tandem. Performing an attentional task while maintaining a VWM load often leads to a loss of memory information. These losses are seen even with very simple visual search tasks. Previous research has argued that this may be due to the attentional selection process, of choosing the target item out of surrounding nontarget items. Over two experiments, the current study disentangles the roles of search and selection in visual search and their influence on a retained VWM load. Experiment 1 revealed that, when search stimuli were relatively simple, target-absent searches (which did not require attentional selection) did not provoke memory interference, whereas target-present search did. In Experiment 2, the number of potential targets was varied in the search displays. In one condition, participants were required to select any one of the items displayed, requiring an attentional selection but no need to search for a specific item. Importantly, this condition led to memory interference to the same extent as a condition where a single target was presented among nontargets. Together, these results show that the process of attentional selection is a sufficient cause for interference with a concurrently maintained VWM load.

## Introduction

Attention can be guided in a strategic and efficient manner to perceivable objects in the visual world. How we direct attention is determined by an internal representation of the sought-for stimulus (e.g., Folk et al., 1992). For example, creating a representation of a banana would lead to a visual bias toward items possessing yellow and curved features. It has long been assumed that these representations are stored in visual working memory (VWM) and used as a target template for search (Desimone & Duncan, 1995). However, numerous dissociations have been noted between internal VWM representations and how search behavior is initiated in external arrays.

VWM is a very limited resource that can store goal-relevant visual information for brief durations. VWM can be seen in operation for visual change detection (e.g., Cowan, 2001), mental rotation (e.g., Hyun & Luck, 2007), and various other tasks. Desimone and Duncan (1995) argued that VWM resources are also used to create a mental construct, or template, that could be used to bias the visual system toward target-matching features. The effects of these templates are most directly revealed by memory-driven capture effects. When holding an item in VWM (as part of change detection task), a distractor matching the memory item, appearing in a subsequent visual search task, leads to robust attentional capture (compared to non-memory-matching distractors; Olivers et al., 2006). This suggests that actively retaining information in VWM automatically biases the visual system to be more receptive to matching features. Conversely, attention can be shifted via cues to prioritize certain memory items over others (e.g., Gunseli et al., 2015). These overlapping findings have led to various claims debating the nature of the relationship between the two systems, with, at the most extreme end, that visual attention and VWM should in fact be seen as two processes of the same mechanism (Kiyonaga & Egner, 2013).

Yet, these systems only seem to overlap under very specific circumstances. Carlisle and colleagues (2011) demonstrated that VWM templates were only necessary to guide the visual system to novel target items. The authors observed that the neural marker for VWM (contralateral delay activity [CDA]), via EEG, was observed when participants were primed with a novel search target. Over the course of several repetitions of the same search target, the CDA diminished and then was eventually eliminated. Carlisle and colleagues (2011) proposed that with enough repetitions, or learning, a target template stored in VWM could be transitioned into a long-term memory store (LTM). Intuitively, this makes sense; an active representation of a target does not need to be created in VWM when there is no target variation trial to trial.

VWM templates do not depend just on novelty. Berggren and Eimer (2018) revealed that in dual-target search or when the target item was defined by a complex conjunction of features, a concurrent VWM load led to disruption in visual search, while this interference was not observed when the search was for simple or single items. When VWM interferes with search, it implies that a process within the search uses some of the resources that are otherwise occupied with VWM storage. This appears to depict a clear picture of the relationship between VWM and visual search. When novel or complex information is required to guide attention to a search target, an activated representation of that item is maintained in VWM. Interference then arises when VWM is already loaded with information via the task demand competition (Kiyonaga & Egner, 2013). Searches that are simple and repeated may instead use less effortful LTM representations, which would not lead to the same interference effects.

While this seems to draw a clear divide between VWM and visual search, several interactions between the two systems have yet to be accounted for—namely, when a simple visual search task and VWM continue to affect one another. Woodman and colleagues (2001) revealed that a concurrent VWM load led to slower visual search response times (RTs). These visual searches were for a specific repeated shape. However, the visual load did not affect the slope of the set size function but caused a general RT slowing (whereas retaining a spatial working memory load led to less efficient search; Woodman & Luck, 2004). Furthermore, here and in several other studies, it has been noted that completing attentional tasks within a VWM retention period leads to lower change detection performance (Hamblin-Frohman & Becker, 2019; Tas et al., 2016; Woodman et al., 2001).

Change detection tasks map the different stages of a VWM event. When items are first displayed, they must be encoded into memory; this rate of encoding is fast but limited (Bays et al., 2011). Next, the items are consolidated into memory, transferring them from brief sensory traces to more robust representations (taking ∼50 ms per item; Vogel et al., 2006). In the maintenance phase, the items are stored, waiting for use. This maintenance phase is not static storage, however; allocation of memory resources can be shifted to prioritize memory precision for certain items (Bays et al., 2011) at the cost of other uncued items (LaRocque et al., 2015). Finally, at test, the memory items must be retrieved to make a change detection judgment to the returning memory array. The interference effects that are discussed here arise from attentional tasks presented within the maintenance phase of memory. It is unlikely that, given sufficient time, attentional tasks interfere with the encoding or consolidation period. Furthermore, Fougnie and Marois (2009) revealed that interference within dual-VWM tasks was unlikely to be attributed to the retrieval process but instead was due to competition during the maintenance phase. VWM storage capacity is highly limited (e.g., Cowan, 2001). If we consider this capacity as a resource, then when memory is already at capacity and additional resources must be used (e.g., adding more items to memory or performing a secondary task), then competition for the limited resources will ensue (Kiyonaga & Egner, 2013). Conversely, when memory capacity is not full, tasks that would normally cause interference do not impact change detection performance (Hamblin-Frohman & Becker, 2019).

Woodman and Luck (2010) explored the impact of visual search on VWM retention effect by varying search requirements in an attempt to identify what process in search led to the loss of memory information. Over three experiments, the authors revealed that interference was not due to a preparation strategy, target selection, or response generation. This led the authors to claim that the visual search interference was due to a nonspecific masking effect, where the presentation and interaction with visual items inadvertently led to accidental search item encoding into VWM. However, some unaccounted-for processes could explain search's impact on VWM retention. Hamblin-Frohman (2022) tested a variety of components in visual search in an attempt to identify which was responsible for the loss of VWM information. The author ruled out several components as the source of interference. Eye movements, spatial-attention shifts, nontarget variance, and target salience all had no impact upon the interference effect. Interference was, however, not observed when only a single item was presented to respond to (Experiments 2 and 3) or when all of the search items in the display were identical targets (Experiment 5). A key commonality between the attentional tasks that provoked interference was the presence of nontarget items. The author concluded that the interference effect was likely due to a selective attentional component, of choosing the target stimulus out of the nontargets.

Selective attention is the act choosing an item out of its environment to attend to for further processing at the expense of the unselected items (Gazzaley & Nobre, 2012). Luck and Hillyard (1994) noted that the N2pc (an EEG marker of attentional selection) only occurred for target selection when it was presented among nontarget items. The neural marker was not elicited when the target was presented alone or when several target items were presented simultaneously. This seems to lend support to Hamblin-Frohman's (2022) claim that there is a distinct process for selection of a target out of nontargets, compared to attending a sole target. This act of selection may lead to the automatic encoding of the selected stimulus (or its features; Marshall & Bays, 2013) into VWM, causing contamination or competition with maintained information.

The current study extends the findings of Hamblin-Frohman (2022) and Woodman and Luck (2010) by identifying whether attentional selection is a sufficient cause for interference with concurrently maintained VWM information. To that aim, it is necessary to experimentally differentiate the *search* and *selection* processes of visual search*.* If the selection hypothesis is correct, then no interference should be observed when only the “search” portion of visual search is completed. However, interference should still be observed when a target is selectively attended even if no search had been performed. Conversely, if interference arises due to a mechanism in the search process, then interference should be observed whenever a search is performed, even if no target selection occurred.

## Experiment 1

Experiment 1 investigated the impact of visual search *with* target selection, compared to visual search *without* selection, upon a retained VWM load. This question has been previously assessed by Woodman and Luck (2010), where target-present search trials (search and selection) were compared to target-absent trials (search and no selection). The authors found no differential impact on VWM performance between target-present and target-absent trials. However, their design used a difficult search with highly similar target and nontarget items. Participants searched for a Landolt-C–like square that was oriented upward or downward out of surrounding Landolt squares oriented to the left or right. Positive search slopes were observed, with RTs increasing with search set size, implying that the search was effortful (e.g., Treisman & Gelade, 1980). This leads to potential additional differences between target-absent and target-present trials besides the process of target selection. Target-absent trials could require a final verification stage with a comparison between a target representation and the stimuli viewed within that trial (Chun & Wolfe, 1996). Furthermore, the stimuli sharing multiple features with the target item (only varying in orientation) may have led to the need to make comparisons with an active representational template of the target stimulus, thus encroaching upon VWM resources. It is plausible that the additional difficulty from the stimuli used in these searches led to a baseline interference effect when compared to the no-task control trials. For these reasons, Experiment 1 replicated Woodman and Luck's (2010) difficult search stimuli and compared them against an easy to discriminate pop-out search task.

Attentional tasks were presented within the retention period of a change detection task: Participants either searched for a specifically oriented Landolt square among other Landolt squares or, in a simple version, searched for a triangle presented among nontarget diamonds. On 50% of trials, the target was absent, with no response or target selection process necessary. Furthermore, to assess whether there is an additional cognitive cost of making an “absent” decision, a condition was included where only a single item was presented at fixation. The participant's task was to report the orientation of the target stimulus if it was present or to do nothing if the target was absent.

If interference is due to the attentional selection component of visual search, then additional interference should be observed on target-present visual search trials and not in target-absent search trials, compared to their respective single item conditions. Due to the additional task requirements for the complex stimuli, this effect should only emerge for the simple stimulus condition. Contrarily, if interference from visual search trials is initiated through the process of *search* and not *selection*, then interference should be observed to the same extent in target-absent search trials as target-present search trials for both complex and simple stimulus conditions.

### Methods

#### Participants

To estimate required sample size, the interference effect observed for a similar display type in Hamblin-Frohman (2022) was used (Experiment 5: high competition condition, *t*(23) = 3.26). To achieve a power of 90% (with 50% assurance), the BUCSS tool suggested a planned sample size of 30 (Anderson et al., 2017). Thirty-five paid participants from the University of Queensland participated in the experiment. After exclusions (detailed below), 33 participants were left for the final data analysis (*M* age = 23.7 years, [*SD* = 3.1], 23 female). The study was granted approval by the University of Queensland ethical board.

#### Apparatus and stimuli

Stimuli were presented on a 17-in. CRT monitor (refresh: 60 Hz). The experiment was controlled by Psychopy in Python language (Peirce, 2007). Stimuli were presented against a gray backdrop. The memory stimuli consisted of four colored squares (height: a visual angle of 1.72°). The memory items were 3.50° away from the center of the screen at cardinal points, where a fixation cross was rendered (height: 0.29°). The memory items had seven potential equiluminant (32 ± 2 cd/m^2^) colors (in RGB: Red [255, 92, 107], Orange [234, 119, 58], Gold [188, 147, 39], Green [90, 171, 116], Blue [49, 160, 209], Purple [165, 131, 217], and Pink [231, 100, 179]). Memory colors were randomly selected on every trial. On “change” trials, a single item would change to an unused color.

The attentional task was completed in the retention period of the memory task. In the complex condition, the search stimuli were all squares (1.15° × 1.15°) with a gap (0.57°) in the middle of one side. The target had either a gap on the top or bottom side, while the nontargets had the gap on the left or right. In the simple condition, the nontargets were diamonds (1.91° × 1.91°), and the target was a triangle (height: 1.43°, width: 1.72°). All attentional task stimuli were unfilled shapes with a black outline. In the single-item condition, the stimulus was presented at fixation. In the search condition, six stimuli were presented (either all nontargets or five nontargets and one target) equidistantly on the outlines of an imaginary circle centered at fixation (radius: 8.10°).

#### Procedure

**Figure 1.**
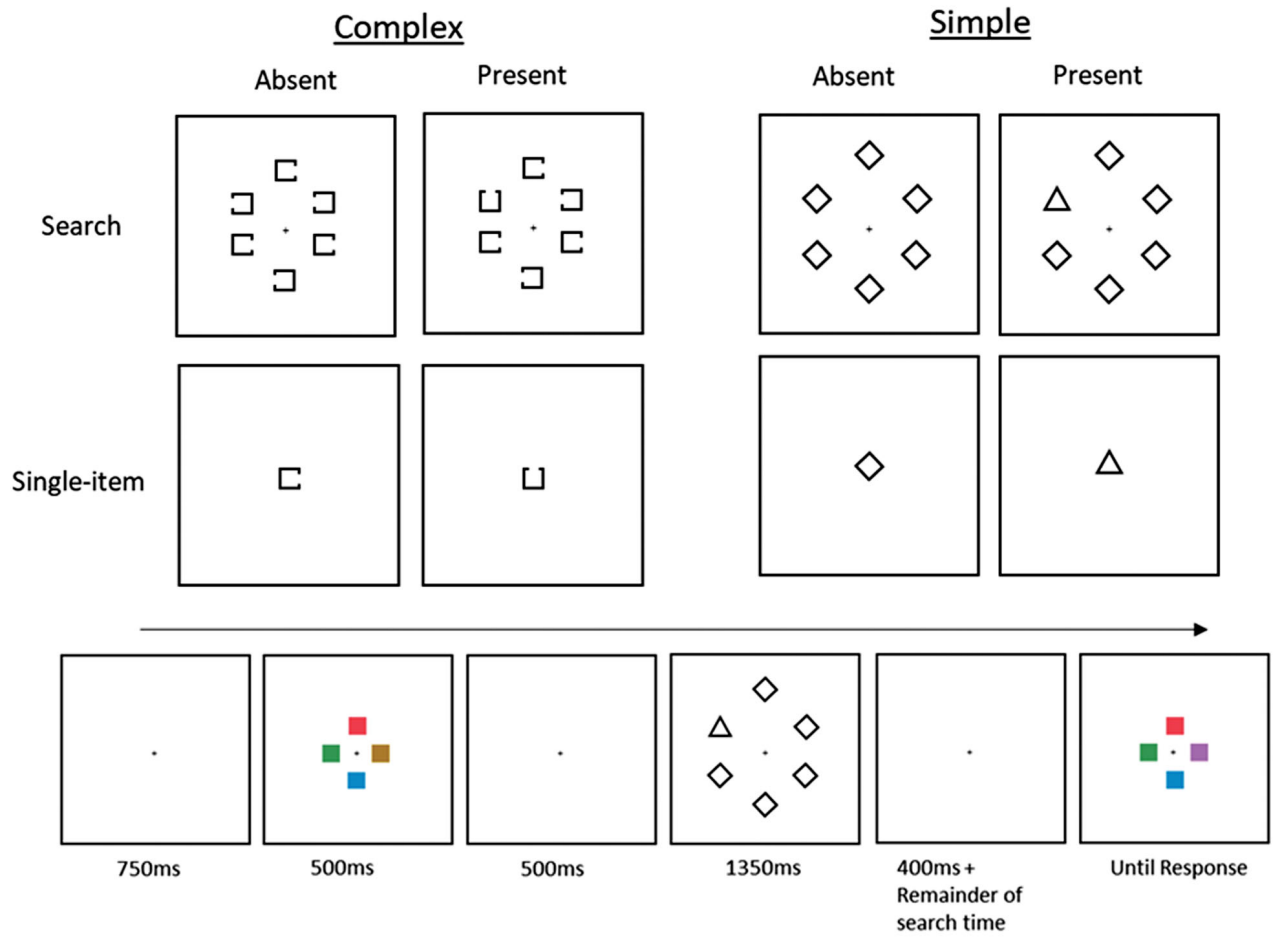
Above: Secondary-task displays used in Experiment 1, presented in the retention period of the change detection task. Participants either responded to the orientation of the upward- or downward-facing target item or did not make a response if the item was absent. On the left are the Landolt squares used in Woodman and Luck (2010) used for the complex-item condition. In the simple-item condition on the right, the nontargets were diamonds with a target triangle. In the search condition, there were always at least five nontarget items, requiring participants to *search* for the target and then make an orientation judgment or no response if absent. In the single-item condition, only a single stimulus was presented, to which participants made the same decisions. Below: The trial structure of Experiment 1 (and Experiment 2). Participants encoded the briefly displayed colored squares and then performed the attentional task presented in the retention period. After completion of the search task, the memory display returned until participants made a “same” or “different” keyboard response.

The memory encoding array was presented for 500 ms. After an additional 500-ms blank screen elapsed (ensuring that the memory display was fully consolidated; Vogel et al., 2006), the attentional task was presented. The task array was presented for 1,350 ms. Participants were instructed to search for the specific target (either the square with a gap on the top or bottom or the triangle, dependent on block) and respond to its orientation. On target-absent trials, participants were instructed not to make a keyboard response. If a response was made on target-present trials in that time the array was removed, if this was on a target-absent trial, the trial was aborted and feedback was displayed. For target-present trials, if a response was not recorded within 1,350 ms, the trial was cancelled and feedback was displayed. After an additional 400 ms, the memory array returned. Participants made a change (*d* key) or no-change (*s* key) judgment for the change detection task, to report whether the test items were identical to or different from the memory items. For the no attentional task *control* trials, the retention period was the same as the attentional task trials (a total of 1,750 ms), but no stimuli appeared, and the participants were not required to make any responses. After a memory response was recorded, there was an additional 750-ms delay before the next trial commenced. Condition displays and trial procedure can be seen in Figure 1.

#### Design

Trials were divided into four discrete blocks. The attentional task was either a visual search with six stimuli presented or a single-item condition (presented at fixation). For each task condition, there were blocks with either the simple stimuli (diamond nontargets and triangle target) or the complex item conditions (differently oriented Landolt squares). The order of blocks was randomly counterbalanced across all participants. In each block, there were 20 control trials (80 total across all blocks), with no attentional task presented. Of the 80 task trials within each block, 50% were target-present and 50% were target-absent trials. This led to a total of 400 experimental trials. For the memory task, on 50% of all trials, a single item changed color, and on the other 50%, all remained the same. Ten practice trials were completed prior to the experiment.

### Results

#### Data

Target-present trials with an incorrect response (1.6%) and target-absent trials where a response was made (1.5%) were excluded from the data. Memory responses greater than 4,000 ms were also excluded (2.7%), and 4.3% trials were lost from a failure to respond to target-present trials in time. Two participants were excluded for failing to reliably complete the target-present searches (> 60% trials excluded for the complex search task). All data are available at https://osf.io/etjsn.

#### Simple-item condition

**Figure 2.**
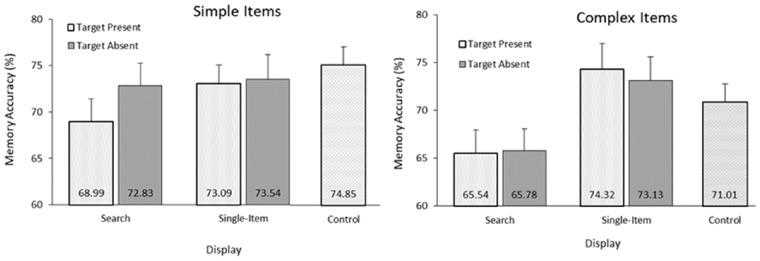
Memory accuracy results from Experiment 1. In the complex-item condition, no effects of target presence were observed; the only effect upon VWM performance was the addition of the search component in the displays, which led to lower accuracy when compared to the corresponding single-item conditions. In the simple-item condition, the results pattern changed: Target-absent trials with a search component did not lead to lower accuracy (when compared to the single-item trials), but target-present trials did lead to lower accuracy. Error bars represent within-subject confidence intervals as according to Loftus and Masson (1994).

To test for general VWM interference effects, each condition was compared against the control trials (see Figure 2 for condition means). As predicted by the *selection* hypothesis, only the target-present search trials led to lower memory performance, *t*(32) = 3.10, *p* = 0.003, BF_10_ = 11.57, compared to the control trials. There was weak evidence for no difference between the control and the target-absent trials, *t*(32) = 1.13, *p* = 0.266, BF_10_ = 0.34, and satisfactory evidence for no difference between the single-item conditions and the control *t*s(32) < 0.68, *p*s > 0.502, BF_10_s < 0.23.

Comparing within the simple-item conditions, a 2 (Display: Search, Single-item) × 2 (Target: Present, Absent) repeated-measures analysis of variance (ANOVA) was conducted on change detection accuracy. Results revealed a trending main effect of display type, *F*(1, 32) = 3.59, *p* = 0.067, ƞ^2^_p_ = 0.10; a trending effect of target presence, *F*(1, 32) = 3.88, *p* = 0.058, ƞ^2^_p_ = 0.11; and a trending interaction, *F*(1, 32), = 3.46, *p* = 0.072, ƞ^2^_p_ = 0.10. Planned two-tailed *t*-tests revealed that for target-present trials, the search display led to lower memory accuracy than the single-item display, *t*(32) = 2.71, *p* = 0.011, BF_10_ = 4.07, while for target-absent trials, there was no difference in memory performance between the two display types, *t*(32) = 0.37, *p* = 0.717, BF_10_ = 0.20, revealing that the addition of the search array was only costly when the search target was present. When comparing between display types, target-present searches led to lower accuracy than target-absent search trials, *t*(32) = 2.87, *p* = 0.007, BF_10_ = 5.76, while there were no differences between present/absent responses in the single-item displays, *t*(32) = 0.17, *p* = 0.870, BF_10_ = 0.19, revealing that the cost of the target presence was unique to the search trials specifically.

#### Complex-item condition

In the complex-item condition, both target-absent and target-present search trials led to lower memory performance than the control (no attentional task) trials, *t*s(32) > 2.94, *p*s < 0.006, BF_10_s > 6.67. This differed from the simple-item condition (where only target-present trials interfered) but replicated the effects seen in Woodman and Luck (2010). Potentially, selection processes may have occurred for the more complicated (and target-similar) nontargets, to confirm the absence of the actual target. Alternatively, the overall complexity of this task may have led to a more general dual-task interference effect. There was anecdotal evidence for a null result between the control single-item condition trial types, *t*s(32) < 1.40, *p*s > 0.170, BF_10_s < 0.46, but numerically, accuracy was in fact higher for the single-item task than the controls.

To compare within the complex-item conditions, a 2 (Display: Search, Single-item) × 2 (Target: Present, Absent) repeated-measures ANOVA was conducted on change detection accuracy. A main effect of display type emerged, *F*(1, 32) = 31.03, *p* < 0.001, ƞ^2^_p_ = 0.49, but no effects of target presence, *F*(1, 32) = 0.18, *p* = 0.679, and no interaction, *F*(1, 32), = 0.12, *p* = 0.729. Planned comparisons revealed that the search display led to lower memory accuracy than the single-item display for both target-present, *t*(32) = 3.92, *p* < 0.001, BF_10_ = 67.13, and target-absent trials, *t*(32) = 4.26, *p* < 0.001, BF_10_ = 159.59. When comparing between display types, there were no differences between target-present and target-absent trials for both search displays, *t*(32) = 0.04, *p* = 0.967, BF_10_ = 0.19, and single-item displays, *t*(32) = 0.53, *p* = 0.601, BF_10_ = 0.21.

### Discussion

Experiment 1 revealed a differing results pattern between target presence and stimulus complexity upon VWM interference. In the simple-item condition, a decrease in memory performance was observed for target-present searches compared to target-absent search trials. Importantly, memory performance decreased for target-present search trials compared to target-present single-item trials, whereas there were no differences between target-absent search and single-item trials. The only condition containing attention selection was the target-present search trials, and this was the sole condition where interference was observed compared to the control trials.

This pattern of results did not extend to the complex-item condition. Here it was observed that both target-absent and target-present search trials interfered to the same extent, when compared to both the respective single-item conditions and control trials. These results replicated the findings of Woodman and Luck (2010) and support the interpretation that more complex search tasks (which may require comparing the stimuli to active templates) will lead to VWM interference regardless of selection requirements. Another explanation for this is that in the target-absent trials, participants selected the nontargets (due to their high similarity) to confirm that they were not target matching.

## Experiment 2

Experiment 1 revealed that target-absent search trials did not necessarily lead to interference, at least for simple stimuli. However, it could be argued that in the simple-item search condition, target presence was easily perceivable and that this resulted in no need to *search* for the target in absent trials, resulting in a condition where participants could dedicate full allocation of resources to VWM. To further corroborate these results, Experiment 2 isolated the selection process from search by varying the number of potential target items in each display. In three blocked, attentional task variations, three stimuli were presented in the retention period of a change detection task. In each task, participants were required to report the orientation of the gap for one of the presented Landolt squares. In the *nontarget condition*, a single Landolt square was presented along with two closed, nontarget squares, which required both a search and a selection process. In the *varied-target condition*, three targets were presented but with all different orientations. This required participants to select an item out of the three but with no need to search or be guided to a specific item. Finally, in the *identical-target condition*, all three items were targets, and all had the same orientation, requiring neither a search nor a selection process.

### Methods

#### Participants

Thirty-three paid participants from the University of Queensland participated in the experiment for a planned sample of 30. After exclusions (detailed below), 29 participants were left for the final data analysis (*M* age = 23.7 years [*SD* = 3.1], 21 female).

#### Stimuli and procedure

The stimuli used were the same as those from Experiment 1 with the following differences. In all conditions, three stimuli were presented. These three stimuli were presented at random locations from the six-stimuli search array used in Experiment 1. In the identical-target condition, three Landolt squares were presented with the same gap orientation (left, right, up, or down). In the varied-target condition, three Landolt squares were presented with all differing orientations. In the nontarget condition, two of the Landolt squares were replaced with closed squares, with the remaining having any of the four orientations. In all three conditions, participants were required to report the orientations of the Landolt square gaps using all four keyboard arrow keys. In the identical and nontarget conditions, there was only one possible correct response; in the varied-target condition, participants were instructed to select a single square and respond to it, leaving three potential correct responses and one incorrect response. All trial timings were the same as Experiment 1, but the attentional task duration was now only 1,150 ms (from 1,350 ms) due to the reduction in task complexity and search array size. In each block (all randomly counterbalanced), there were 108 attentional task trials with 54 no-task control trials intermixed, for a total of 486 experimental trials. Participants completed 10 practice trials before commencing the main experiment.

### Results

Trials with incorrect search responses were excluded from analysis (2.6% or trials). Furthermore, four participants were excluded for having less than 85% search accuracy in the varied-targets condition (where chance performance was 75%). Memory responses greater than 4,000 ms were also excluded (2.1%). Of the trials, 1.1% were lost from a failure to respond to the attentional task in the time window.

**Figure 3.**
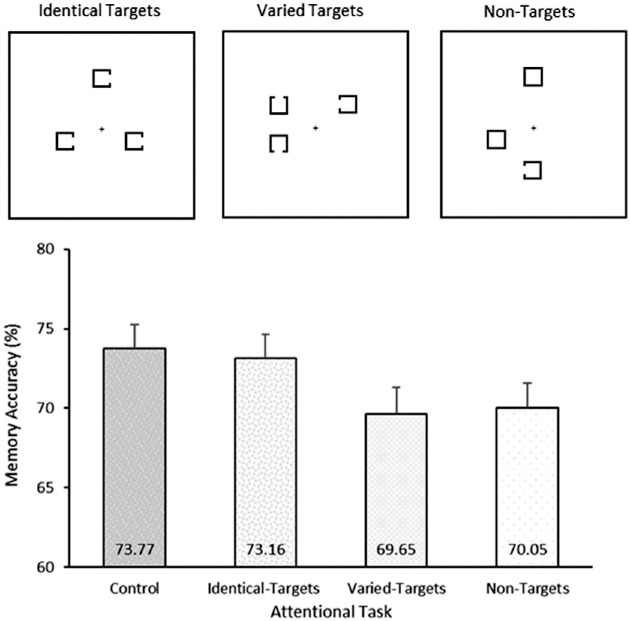
Above: Attentional task displays used in Experiment 2. Participants were instructed to respond to the orientation of the Landolt-Cs presented. In the identical-target condition, three items were presented all with the same orientation. In the nontarget condition, two of the squares were filled, leaving only a single item to respond to. In the varied-target condition, three items were presented with all different orientations, and participants were instructed to respond to any of the three. Below: Results from Experiment 2. Interference (compared to the control trials) was observed for both the varied and the nontarget conditions but not for the identical-target condition. Accuracy was also significantly higher for the identical targets than both other attentional task conditions. Error bars represent within-subject confidence intervals as according to Loftus and Masson (1994).

To test interference effects, a one-way repeated-measures ANOVA (control, identical targets, varied targets, nontargets) was conducted on memory performance for the change detection task. A significant effect emerged, *F*(3,87) = 6.17, *p* < 0.001, ƞ^2^_p_ = 0.18, displayed in Figure 3. Planned two-tailed comparisons revealed that as predicted, memory accuracy was significantly higher for the control trials compared to the nontarget condition, *t*(28) = 3.22, *p* = 0.003, BF_10_ = 11.88. Furthermore, when the search component was removed in varied-target condition, memory interference was observed (compare to the control), *t*(28) = 3.41, *p* = 0.002, BF_10_ = 18.32. Importantly there were no observable differences between the nontarget condition (search and selection) and the varied-target condition (selection alone), *t*(28) = 0.36, *p* = 0.740, BF_10_ = 0.21. The identical-target condition had higher accuracy than the varied-targets condition, *t*(28) = 2.95, *p* = 0.006, BF_10_ = 6.65, and the nontarget condition, *t*(28) = 2.36, *p* = 0.025, BF_10_ = 2.11, and did not differ from the control trials, *t*(28) = 0.54, *p* = 0.540, BF_10_ = 0.23. This reflected that the display characteristics and response were not responsible for the interference effect observed in the other conditions.

### Discussion

Experiment 2 disentangled the *search* and *selection* components of visual search in a novel manner and revealed that the selection mechanism impacted the retained VWM information even when the search components was removed. In the nontarget condition, participants were required to both search for the target and then select it from the nontargets. Importantly, the interference seen in this condition was equivalent to the varied-target condition where a single item was selected without the requirement to search. A potential consideration for the varied-target conditions was that participants would seek out a specific orientation to respond to, for example, searching for the *upward* oriented item on each trial, leading to search within this condition. This, however, would be a more effortful task for participants than responding to the first item selected and would only be a valid strategy on 75% of trials. Unfortunately, this explanation cannot be ruled out, but it is viewed as an unlikely account for the observed results. Together, both the varied and nontarget conditions interfered with memory compared to the no-task controls and the identical-target condition. With the common factor between both interfering conditions being attentional selection, it suggests that this selective process is the sufficient factor for VWM interference.

## General discussion

A growing body of evidence highlights the divide between mental representations and how attention is in fact guided throughout our visual environment (Hamblin-Frohman & Becker, 2021; Wolfe, 2021; Yu et al., 2022). Even when feature-specific information is stored for a target stimulus, guidance rules unrelated to this can be the primary factor in determining how attention is initially allocated in a display (Hamblin-Frohman et al., 2023). This divide between memory and attention appears quite apparent when looking at how VWM affects visual search, yet the impact of visual search on VWM is less clear.

The current study aimed to identify why a visual search task causes information loss for concurrently retained VWM information. Both experiments aimed to dissociate two processes of a visual search task: the act of searching, and guiding attention, throughout a display to locate a target and the act of selecting that target as the sought-for item. In Experiment 1, the search process was isolated from the target selection process. Target-absent trials were used as a method to create search trials with no selection process required. Critically, it was observed that (for simple stimuli) the search component only led to interference when there was a target contained within the display (requiring selection). In the target-absent trials, no such target selection was required, but the display still required scanning to determine its absence. In Experiment 2, the requirement to search for a specific stimulus was removed from the attentional tasks. When participants were required to select one target item out of other target items, interference was observed. Comparatively, when these targets all contained identical information (no selection and no search were required), no interference was observed. Together, the findings across the two experiments strongly suggest that attentional selection is a sufficient cause for memory interference to occur.

Within the process of selecting the target item, there are several underlying mechanisms. Previously, in Woodman and Luck (2010) and Hamblin-Frohman (2022), a few of these were eliminated as potential interference components. Physical response generation, stimulus–response mappings, and target gaze fixations could not account for memory interference. Woodman and Luck (2010) varied the need to make physical responses and found no variations in memory performance, while in the current study, responding to single-item displays (Experiment 1) and the identical targets (Experiment 2) led to no interference effects when compared to baseline controls. One consideration is that the competition between the stimulus signals of targets and nontargets could account for the interference effect. Desimone and Duncan (1995) described selective attention as emerging from the resolution of competing stimulus signals. The interference effect in turn may be due to the competition between signals and not the selective process itself. In Hamblin-Frohman (2022; Experiment 5), target discriminability was manipulated, with the nontargets being reduced to small dots on the screen (compared to a target triangle). Even in the highly discriminable target condition, memory interference was observed to the same extent when compared to a less discernable search target. This suggests that the strength of the competition itself is not the primary factor in determining whether VWM interference occurs. However, when competition is very high between target and nontargets, the selection process may become more strenuous, leading to additional depletions of memory resources (e.g., the complex-item condition in Experiment 1).

As noted by Luck and Hillyard (1994), the N2pc only arose for target selection when nontarget items were present, and later, Eimer (1996) interpreted this as the N2pc being indicative of attentionally selecting the target stimulus. As the visual system is hierarchical (e.g., Hochstein & Ahissar, 2002), it is plausible that selective attention automatically stores the attended stimuli at a representational level that is (or uses) the same resources used for VWM maintenance. For instance, Marshall and Bays (2013) noted that irrelevant features of selected items would be automatically encoded into VWM, interfering with retention for relevant information. This could well explain the current findings; the selected search targets (or their features) were obligatorily encoded into VWM, replacing or weakening the representations of the stored items. Alternatively, previous work has identified that attentional allocations can be shifted from one memory representation to another (e.g., LaRocque et al., 2015). The effects seen here seem to mimic that, when a perceived item is selectively attended, some of the allocations toward storage have instead been directed externally to the percept. Even though the item may not have been encoded into memory, the memory resources loss from performing the selection could have led to the lower change detection performance. Either proposal, encoding of the search target or memory resource depletion, could explain the observed interference effects. Importantly, these effects should only be observed when the combined needs of both the memory and attentional task exceed the VWM capacity limits (Hamblin-Frohman & Becker, 2019; Kiyonaga & Egner, 2013).

It should be noted that the current experiments do not provide evidence that attentional selection is the *only* component that can interfere with VWM. In the complex-item conditions of Experiment 1, clear interference was observed for target-absent trials. These trials were of course much more demanding than the target-absent trials in the simple-item conditions. The search itself was more complex, and the nontargets were much more target-similar than those in the simple-item condition. This highlights that interference is not strictly limited to attentional selection.

A few studies have shown that single stimulus presentations within a VWM retention period do not lead to a loss of memory information (Hamblin-Frohman & Becker, 2019; Tas et al., 2016). In these instances, this key selection process does not occur, thus providing an explanation for the uncorrupted memory. Yet in real-world visual environments, there are rarely moments when only a single item is available for selection. This means that items stored in VWM are most likely under constant threat when concurrently interacting with the visual world. As described by Olivers and colleagues (2011), a single item in memory may be elevated to a higher activation state than other less important items and may receive shielding from external visual interference. This would allow both attentional and memory tasks to be completed in tandem but at the cost of the amount of information processed in either domain. But, as in the current experiments, when VWM capacity is fully loaded with equally important objects, it seems impossible to fully protect memory from selective attentional interference.

Recent studies requiring participants to gather and then reproduce information have revealed that these more naturalistic settings lead to VWM not being used at full capacity (Draschkow et al., 2021). Instead, behavior follows the slower method of relying on externally available information instead of multiple internal representations (Somai et al., 2020). This appears to be an adaptive response to the potential cross-task interference. VWM operates at a reduced capacity to provide a shielded internal representation preventing the memory contents from being disrupted by selective attention occurring in the attentional task. Further studies examining how VWM adapts to protect for real-world search arrays and the limits to the protection it can afford may further expand on the link between selective attention and visual memory.
